# Glassy Drug Microneedle Array Design: Drug Glass-Forming Ability and Stability

**DOI:** 10.1021/acs.molpharmaceut.4c01067

**Published:** 2025-02-17

**Authors:** Mohamed Elkhashab, Ziad Sartawi, Waleed Faisal, Abina Crean

**Affiliations:** † SSPC, the Research Ireland Centre for Pharmaceuticals, School of Pharmacy, University College Cork, Cork T12 K8AF, Ireland; ‡ ArrayPatch Ltd., Euro Business Park, Little Island, Cork T45 FX94, Ireland

**Keywords:** glassy microneedles, stability, glass-forming ability, glass transition temperature, humidity, hygroscopicity

## Abstract

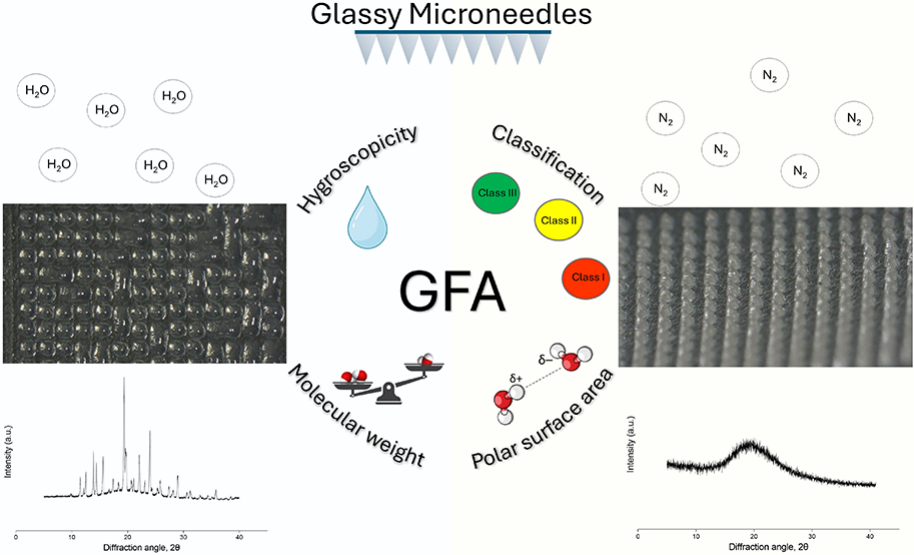

Glassy microneedles, composed only of drug, provide an intradermal alternative to oral or parenteral drug delivery. Compared to microneedles composed of drug–polymer solid dispersions, they offer higher drug loading while possessing mechanical strength for skin penetration. However, their microneedle structure and associated mechanical strength are reliant on the component glass stability. This study investigates relationships between the glass stability of drug-only microneedles and drug glass-forming ability (GFA), determined by differential scanning calorimetry (DSC) analysis. The glass stability of microneedles fabricated from six drugs was evaluated at 2–8 °C under nitrogen, 25 °C/60% relative humidity (RH), and 40 °C/75% RH. Drug glass stability was determined by visual assessment of microneedle appearance, together with DSC and powder X-ray diffraction analysis of the drug melt cooled outside the microneedle molds. Glassy microneedle structure was retained for all drugs stored at 2–8 °C under nitrogen for 3 months. Drug GFA classes informed glass stability under dry (nitrogen) environments at temperatures below their glass transition temperature. Under controlled humidity conditions, all glass microneedles crystallized, except for itraconazole. Drug GFA did not inform microneedle glass stability when exposed to water vapor during storage due to water absorption and glass plasticization. Itraconazole’s glass stability was attributed to the interaction of absorbed water with liquid crystalline phases present in the itraconazole glass. The results highlight how glassy microneedle stability is informed by storage below *T*_g_ and glass interaction with moisture vapor. Results also demonstrate how the skin penetration efficiency of glassy microneedles is maintained during storage by selecting stabilizing storage conditions.

## Introduction

Microneedle arrays incorporating drug molecules represent a safe and efficient method for intradermal drug delivery to treat skin diseases or conditions. As an alternative to traditional oral or injection-based drug delivery methods, the microneedle drug delivery system can offer greater efficiency compared to current topical skin treatments.^1^ Microneedles do not reach sensory nerve endings present in the skin; hence, they are pain-free, relatively easy to administer, and therefore have the potential for good patient compliance.^2^ Researchers have explored various types of microneedles, including solid, hollow, coated, and dissolvable microneedles.^3−5^ However, there has been a recent surge in interest in dissolvable microneedles, both in academic research and for commercial exploitation.^3,6^ This trend is attributed to the ease of fabrication and disposal of the dissolvable microneedles, as well as the fact that they do not require a separate delivery device, unlike hollow microneedles. This design facilitates more efficient drug release and dissolution within the skin layers.^5,7−10^

Dissolvable microneedles have the potential to incorporate drug doses higher than those of coated microneedles. Despite this relative advantage, delivering a high-dose drug remains a challenge for dissolvable microneedles.^11,12^ The loading of drugs into dissolvable microneedle arrays is typically diluted by matrix polymer and other excipients included to increase microneedle mechanical strength for skin penetration.^6^ An advantage of dissolvable microneedle structures is their capacity to incorporate drug compounds in a noncrystalline, amorphous solid-state. To date, the application of amorphous drugs in microneedle formulations has focused on their capacity to enhance the solubility and release of hydrophobic drugs compared with their crystalline counterparts. The formation of amorphous drug phases in dissolvable microneedle formulations is predominately achieved via the formation of drug–polymer solid dispersions using solvent casting methodologies. For example, Abraham et al. demonstrated the formation of microneedles containing amorphous rifamycin by embedding the drug in a cellulose matrix.^13^ Woo et al. demonstrated how controlling the amorphous or crystalline state of flurbiprofen, by combining the drug with different ratios of povidone, altered drug release and dermal bioavailability.^14^

The formulation of a drug as a drug–polymer solid dispersion to generate and stabilize a drug’s amorphous state within microneedle formulations substantially limits the microneedle’s drug loading capacity. Azizoglu et al. identified this limitation and explored the preparation of montelukast microneedles without the inclusion of polymer to increase drug loading.^15^ Microneedles were successfully fabricated using both solution casting and powder filling/dissolution methodologies. A powder filling/melting approach was also attempted but failed to produce consistent microneedle structures, resulting in broken needle tips upon removal from molds. The amorphous nature of the montelukast microneedles produced was noted by their transparent visual appearance. Montelukast microneedle arrays demonstrated sufficient mechanical strength for skin penetration, and montelukast was deemed to be a suitable matrix material for microneedle formation. Drug-only microneedle structures have also been fabricated using a novel production methodology, micromolding molten drug into microneedle structures.^16,17^ Itraconazole microneedle arrays produced using this methodology exhibited chemical and physical stability under ambient and accelerated storage conditions, good skin penetration, and dissolved following administration in a porcine in vivo study.^17^

As illustrated by both the montelukast and itraconazole drug-only microneedle structures, the glassy drug form is advantageous for microneedle structures due to its suitability for molding into sharp microneedle structures with sufficient mechanical strength for skin penetration.^15,17^ Formulating glassy drug microneedles for clinical applications requires the drug molecule to exhibit good glass-forming ability and retain glass stability during storage. A limitation of amorphous glasses is their thermodynamic instability, high molecular mobility, and a tendency toward crystallization during storage.^18,19^ Therefore, understanding the crystallization tendency of the glassy drug form is a key consideration in the development of drug-only glassy microneedles for clinical applications.

Numerous studies have elucidated the properties of drug molecules that are good glass formers. Baird et al. introduced a classification system for glass formers employing differential scanning calorimetry (DSC).^20^ The classification categorizes compounds into three classes: class I molecules, which do not form an amorphous state and crystallize upon cooling after melting; class II molecules, which are amorphous upon cooling after melting but recrystallize during heating; and class III molecules, which exhibit an amorphous form upon cooling of the melt and maintain their amorphous form upon heating. An in silico model developed by Mahlin et al. elucidated that larger molecules with low molecular symmetry, fewer benzene rings, branched carbon skeletons, and electronegative atoms are likely to be good glass formers.^21^ The influence of electronegative atoms and hydrogen bonding was shown to influence the formation of loosely packed aggregates that promote the glass state.^22^ Subsequent research classified drugs according to their glass-forming ability (GFA) based on their inherent crystallization tendency, such as their critical cooling rate^23^ or nucleation behavior.^24^

Water absorption by glasses, due to exposure to environmental humidity, plays a significant role in influencing drug glass stability, as absorbed water can increase molecular mobility, reduce the glass transition temperature (*T*_g_), and plasticize glass phases.^25−28^

The study presented builds on studies related to the production of glassy, drug-only microneedle structures introduced earlier.^15,17^ While these studies demonstrated the capability of individual drug molecules to form glassy, drug-only microneedles, in this study, we investigate the effects of controlled temperature and humidity storage conditions on the glass stability of six drug-only microneedles. The study illustrates how prior research exploring drug GFA and glass stability can inform the appropriate storage conditions for glass microneedle structures.^20−27^ Drug-only dissolvable microneedles evaluated include betamethasone dipropionate (BMD) as an anti-inflammatory agent,^29^ indomethacin (IMN) as an analgesic,^30^ estradiol (E2) for hormonal therapy,^31^ itraconazole (ITZ) for antifungal skin infections,^32^ vismodegib (VDG) for skin carcinoma,^33^ and zolmitriptan (ZMT) for migraines.^34^ The glass-forming molecules were selected based on two criteria. First, they are drug molecules with therapeutic indications where local or systemic delivery via intradermal administration may offer clinical advantages. Second, they are chemically stable following melting, rendering them suitable for micromolding their molten form into microneedle structures.

## Materials and Methods

### Materials

Vismodegib (VDG) and betamethasone dipropionate (BMD) were purchased from MedChemExpress (Sweden). Itraconazole (ITZ), zolmitriptan (ZMT), and 17-β-estradiol hemihydrate (E2) were purchased from Kemprotec (UK) Ltd.; indomethacin (IMN) was purchased from Sigma-Aldrich (Ireland) Ltd.; T-zero calorimetry pans were acquired from Waters TA Instruments Ireland (Dublin, Ireland); 1525L adhesive tape was obtained from 3 M, USA; master mold 7000-grade aluminum was provided by SMAE Engineering Workshop, Queen University Belfast (UK) to design a 14 × 14 microneedle array, 500 μm length/height, and 300 μm diameter base; SYLGARD 184 Silicone Elastomer Kit was purchased from Ellsworth Adhesives Limited (UK), and polydimethylsiloxane microneedle molds were manufactured according to the manufacturer’s instructions.

### Drug Glass-Forming Ability Classification

The drug glass-forming ability (GFA) classification employed was based on the previously reported DSC method by Baird et al.^20^ Drug samples were weighed into T-zero aluminum pans and sealed. Samples were purged in a stream of dry nitrogen flowing at 50 mL/min. Samples were heated at a rate of 10 °C/min until reaching approximately 10 °C above their respective melting temperatures and then held isothermally for 3 min. Subsequently, they were cooled at a rate of 20 °C/min to 20 °C, followed by reheating at a rate of 10 °C/min to slightly above the melting temperature. Analysis was performed in triplicate for each drug molecule investigated.

### Fabrication of Microneedles

The microneedle arrays were fabricated according to a methodology previously described.^17^ In brief, the powder of the materials was distributed on plain glass slides and placed in an oven (Vacuum Oven, VACUTHerm, Germany), set at a temperature 5 °C above the melting point of each sample at atmospheric pressure. For microneedle preparation, polydimethylsiloxane microneedle molds were placed on glass slides inside the oven. Once the samples were liquefied, heated molds were placed face down into the molten drug. Oven pressure was reduced to 10 mbar and held for 5 min to fill the molds. Thereafter, the pressure slowly returned to atmospheric conditions. The molds were allowed to cool at ambient conditions, after which the microneedles were extracted from the mold using medical-grade adhesive tape. The manufactured microneedles were stored under different storage conditions detailedin Stability Testing. Control samples of cooled molten drug were prepared (without microneedle molding) and stored under equivalent processing conditions.

Following fabrication, each needle of the microneedle array was visually assessed using an Olympus SZ61 microscope (Philippines) equipped with View 7 software and assigned a score from 0 to 2. Microneedles with perfectly sharp tips received a score of 2, those with nearly perfect, wizard hat, or blunt tips were scored 1, and those with volcano-shaped tips, no tips, or missing needles were scored 0. Microneedle arrays with a score <90% (of the maximum score of 392, (14 × 14 microneedles × 2)) were discarded, while those with a score >90% were accepted for further use.

### Stability Testing

The glass stability of the glassy microneedles fabricated and samples of cooled-molten drug was evaluated in line with ICH/WHO guidelines.^35^ The study was conducted over a 3-month period. Samples were stored under conditions of refrigeration at 2–8 °C, packaged in a sealed vial under nitrogen, 25 °C/60% RH, and 40 °C/75% RH in controlled humidity chambers (Memmert HCP50 Humidity Chamber, Germany). The microneedles and cooled-molten samples were evaluated after preparation and following storage at 1 day, 1 week, 1 month, and 3-month time points under each storage condition. At every time point and for each storage condition, the glass stability of the cooled-molten samples was determined by DSC and PXRD (as described in Amorphous Solid Characterization and Stability Assessment), and microneedles were examined visually by microscope (as detailed in theFabrication of Microneedles). Additional stability studies were conducted to further investigate the stability of ZMT and IMN microneedles when stored at 25 °C in a sealed vial under nitrogen for 3 months. Microneedles were visually assessed under a microscope over a 3-month period.

### Amorphous Solid Characterization and Stability Assessment

The amorphous stability of cooled-molten samples was assessed using DSC and PXRD techniques. The DSC thermogram was recorded using a DSC Q1000 (TA Instruments, UK). Samples were weighed into T-zero aluminum pans (TA Instruments), equilibrated at 25 °C for 5 min, and heated to above the drug’s melting point at a rate of 3 °C/min with an applied modulation of ±1 °C every 60 s. PXRD diffractograms were recorded on a Stadi MP diffractometer (Stoe GmbH, Germany). Quench-cooled samples were irradiated by monochromatized Cu-Kα radiation with a copper X-ray source (λ = 1.5406 Å) at 40 mA and 40 kV. PXRD patterns were collected over the 2θ range of 5–41° at a scan rate of 2°/90 s. The diffraction patterns were analyzed using Philips X’Pert High Score software (version 1.0a).

### Drug Glass Hygroscopicity Assessment

Dynamic vapor sorption (DVS) analysis was used to measure the weight gain of glassy drug samples upon exposure to humidity using the DVS Intrinsic Instrument and DVS Intrinsic Control software (Surface Measurement Systems, UK). This method followed the guidelines of the European Pharmacopoeia.^36^ The samples were placed in the sample chamber, and humidity was set to 0% relative humidity (RH) for an hour; humidity was then increased to 80% for 24 h. Throughout the experiment, the temperature remained constant at 25 °C. Hygroscopicity was determined as follows:
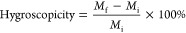
where *M*_f_ is the final mass of the sample at the end of the experiment and *M*_i_ is the initial mass of the sample.

### Skin Penetration Evaluation

To determine the impact of storage on glassy microneedle skin penetration, selected microneedle arrays were evaluated immediately post-manufacture and again after 3 months of storage using the previously reported method.^17^ Neonatal porcine skin, collected from stillborn pigs and stored at −80 °C, was used as a model tissue. After thawing, the skin was prepared by removing cartilage and fat, stretching it over a cork board, and securing it in place. Microneedles in triplicate were applied using an applicator delivering 25 N of force to the array’s center and corners. After removal of the microneedle array, 20 μL of phthalocyanine blue was applied for 5 min and then rinsed off. Skin penetration efficiency was quantified by counting blue marks left at the application site under an Olympus SZ61 microscope with View 7 software. The skin penetration of each microneedle array was evaluated in triplicate.

## Results

### Drug Glass-Forming Ability and Stability

**Figure 1:**
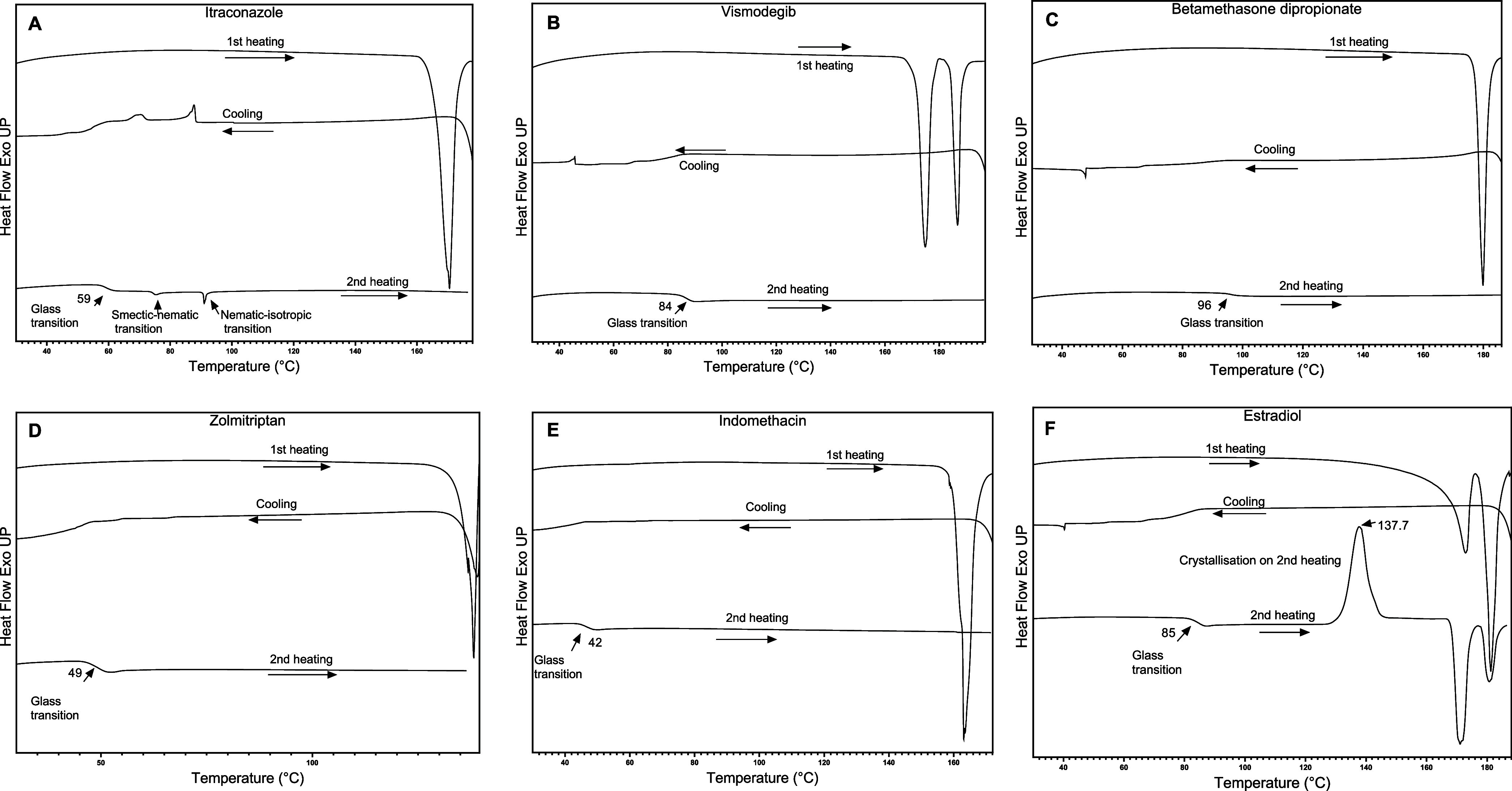
Representative DSC thermograms of (A) itraconazole, (B) vismodegib, (C) betamethasone dipropionate, (D) zolmitriptan, (E) indomethacin, and (F) estradiol (E2) produced during analysis to determine drug glass-forming ability classification according to criteria reported by Baird et al.^20.^

Prior to the formation of glassy microneedles, the glass-forming ability (GFA) of each drug molecule was assessed. Figure 1 presents the DSC thermograms generated for each drug during the heat–cool–heat cycle to determine their GFA class according to the methodology and criteria proposed by Baird et al.^20^ The melting temperature of the starting crystalline form of each drug was determined during the first heating cycle: ITZ (165 °C), VG (180 °C), BMD (178 °C), ZMT (136 °C), IMN (158 °C), and E2 (177 °C). A premelting endotherm at 173 °C was noted for E2 before the melting peak at 177 °C. The premelting peak was attributed to the loss of hydrated water present in crystalline 17-β-estradiol hemihydrate form.^37^ VDG displayed an endotherm at 170 °C prior to the previously reported melting peak around 180 °C.^38^ The first endothermic peak may be related to reported VDG polymorphism.^39^ DSC thermograms of VDG polymorphic forms have not been determined.

For E2, a crystallization exotherm was noted at 137.7 °C, followed by two melting endotherms related to polymorphic Forms I and II.^37^ Endothermic events observed upon reheating the ITZ-cooled melts were identified as thermotropic mesophase transitions (smectic–nematic–isotropic transitions), indicating a glassy structure containing frozen liquid crystalline phases.^40,41^ The *T*_g_ of each amorphous drug phase formed was determined during the second heating cycle. *T*_g_ values ranged from 42 °C for IMN to 98 °C for BMD and were in agreement with previously reported values where available.^42^ To our knowledge, the *T*_g_ values for BMD and VG amorphous phases have not been previously reported.

Of the 6 molecules investigated, only E2 was classified as a class II molecule, displaying good glass-forming ability but poor glass stability. No crystallization or melting events were noted for the cooled melts of the other molecules, which categorizes them as class III molecules, displaying both good glass-forming ability and stability. These findings for ITZ, IMN, ZM, and E2 agree with those previously reported by Baird et al.^20^ and Alhalaweh et al.^42^

### Physical Assessment of the Microneedles

**Figure 2:**
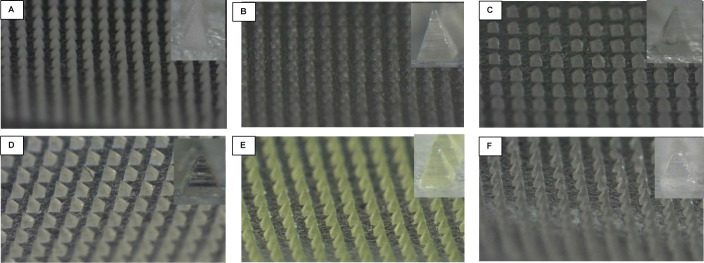
Drug microneedle arrays on the day of manufacturing visualized by an Olympus SZ61 microscope. (A) ITZ array; (B) VDG array; (C) BMD array; (D) ZMT array; (E) IMN array; and (F) E2 array. Inserted images display the structure of individual microneedles.

Each drug microneedle array was visually examined to assess its shape and structural integrity, following the criteria and scoring system detailed in Section 3.3. The needles generally appeared sharp and intact with a glassy, transparent appearance. IMN needles displayed a yellowish transparent color, the color of the IMN powder prior to microneedle fabrication. The ITZ microneedles exhibited a whitish appearance typical of its glassy liquid crystal state, rather than the glassy amorphous state observed with the other compounds.^41^ Visualization of the arrays under the microscope is depicted in Figure 2.

**Figure 3:**
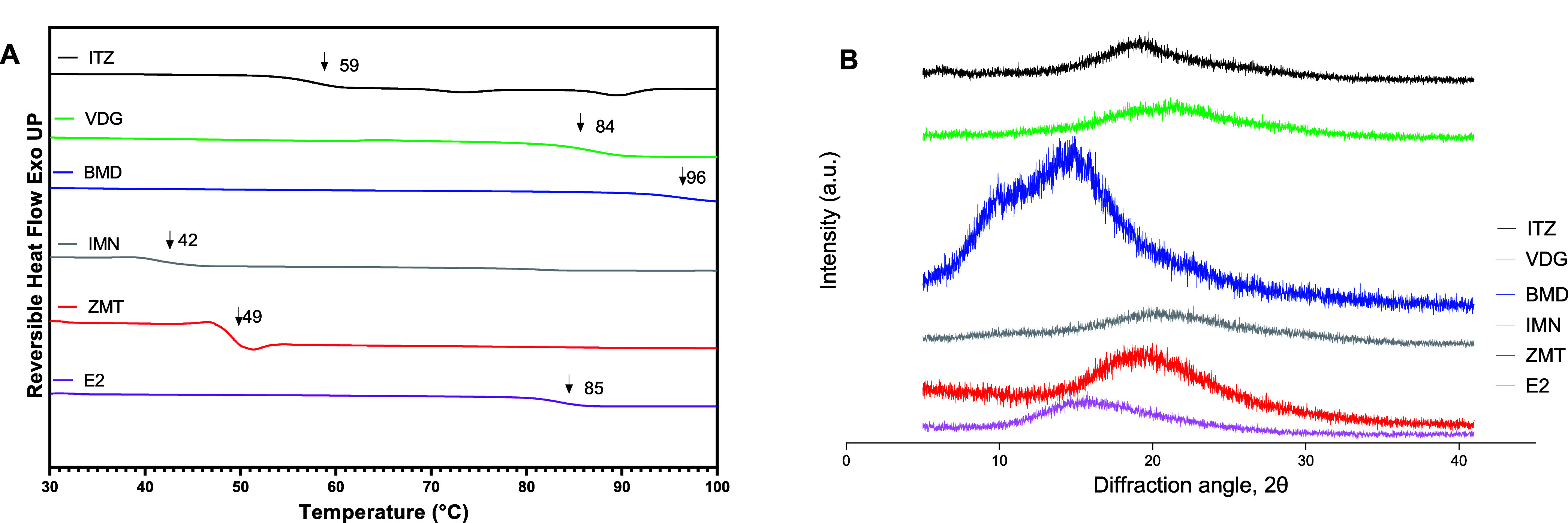
(A) DSC thermogram with glass transition temperatures indicated. (B) PXRD diffractogram of the cooled-molten samples of itraconazole (ITZ), vismodegib (VDG), betamethasone dipropionate (BMD), indomethacin (IMN), zolmitriptan (ZMT), and estradiol (E2).

Due to the dimensions of the microneedle structure and the presence of adhesive after removing needles from the backing tape, it was not practical to verify the glassy state of the microneedles using DSC or PXRD. Therefore, the cooled-molten drug samples of the six drugs were prepared under equivalent conditions to the microneedle preparation and analyzed using DSC and PXRD to determine their GFA and stability. It was observed that all drug substances exhibited a glassy form on the day of manufacture, as illustrated by the DSC and PXRD results (Figure 3). The glass transition temperatures of each drug after preparation of the cooled-molten samples were determined from their respective DSC thermograms (Figure 3A), and the individual *T*_g_ values for each drug are also summarized in Table 1. The absence of crystalline peaks in PXRD diffractograms (Figure 3B) indicates that these solid forms are X-ray amorphous. However, a minor endothermic event at 90 °C in the ITZ DSC thermogram (Figure 3A) can be attributed to a nematic–isotropic phase transition, indicating the presence of liquid crystal phases in the ITZ glass.^40,41^ This was also noted during the GFA classification of ITZ by DSC (Figure 1A).

**Table 1:** Drug Candidates Investigated Including Their GFA Class Determined Experimentally Using DSC Methodology,^20^ Molecular Weight (*M*_w_), and polar Surface Area (PSA) Collected from PubChem,^a^ the Stability of Drug Microneedles and Cooled-Molten Drug Samples under Respective Storage Conditions, Melting Point (*T*_m_), and Glass Transition (*T*_g_) Were Verified by DSC Analysis, and Amorphous Drug Hygroscopicity Determined Experimentally by DVS Analysis

				storage condition and crystallization time point					
compound	GFA class	*M*_w_ (Da)	PSA (Å^2^)	2–8 °C/N_2_	25 °C/60% RH	40 °C/75% RH	*T*_g_ (°C)	*T*_m_ (°C)	hygroscopicity (%)
itraconazole (ITZ)	III	705.7	101	stable	stable	stable	59	165	1.12
vismodegib (VDG)	III	421.3	84.5	stable	stable	crystallized month 3	84	180	0.91
betamethasone dipropionate (BMD)	III	504.6	107	stable	stable	crystallized month 1	96	178	1.44
estradiol (E2)	II	272.4	40.5	stable	crystallized month 1	crystallized day 1	85	177	0.29
indomethacin (IMN)	III	357.8	68.5	stable	crystallized week 1	crystallized day 1	42	158	3.86
zolmitriptan (ZMT)	III	287.4	57.4	stable	crystallized week 1 and melted day 1	crystallized day 1	49	136	6.19

a http://pubchem.ncbi.nlm.nih.gov/.

### Stability Assessment of Drug Microneedle Array and Cooled-Molten Drug Samples

**Figure 4:**
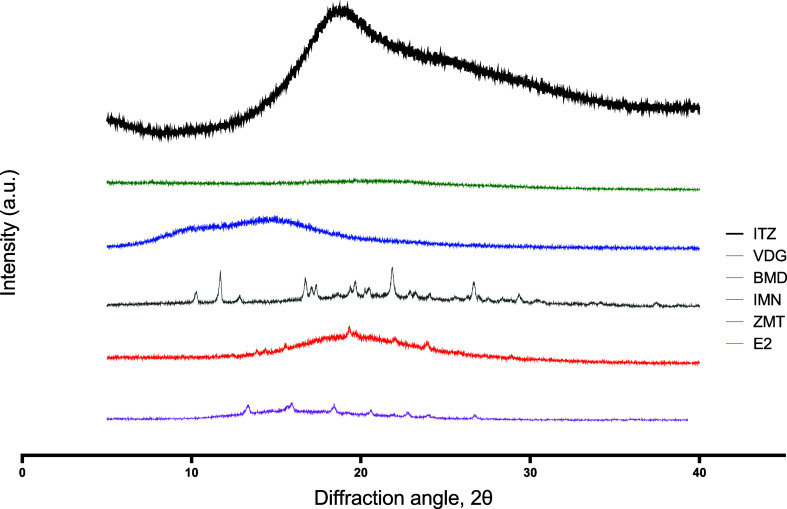
PXRD diffractogram of the cooled-molten samples at the time point crystallization was first observed when stored at 25 °C/60% RH; estradiol (E2) at month 1, indomethacin (IMN) and zolmitriptan (ZMT) at week 1. Itraconazole (ITZ), vismodegib (VDG), and betamethasone dipropionate (BMD) retained their glass stability month 3 under conditions of 25 °C/60% RH with no crystallization peak evident.

Under storage conditions of 2–8 °C with nitrogen, all microneedles and cooled-molten drug samples retained their glassy state throughout the 3-month study period. Microscopic examination revealed no changes in the microneedles’ appearance, the DSC thermograms remained consistent over the 3 months, and no crystallinity peaks were observed during PXRD analysis of the cooled-molten drug samples (Figures S1 and S2). However, at 25 °C/60% RH, the cooled-molten samples of three drugs exhibited glass instability and crystallization: E2 crystallized after one month, IMN melted after 1 week, and ZMT melted on the first day of storage but remained PXRD amorphous for 1 week before showing crystallinity peaks in the PXRD analysis (Figure 4). In contrast, ITZ, BMD, and VDG remained stable at 25 °C/60% RH for 3 months.

**Figure 5:**
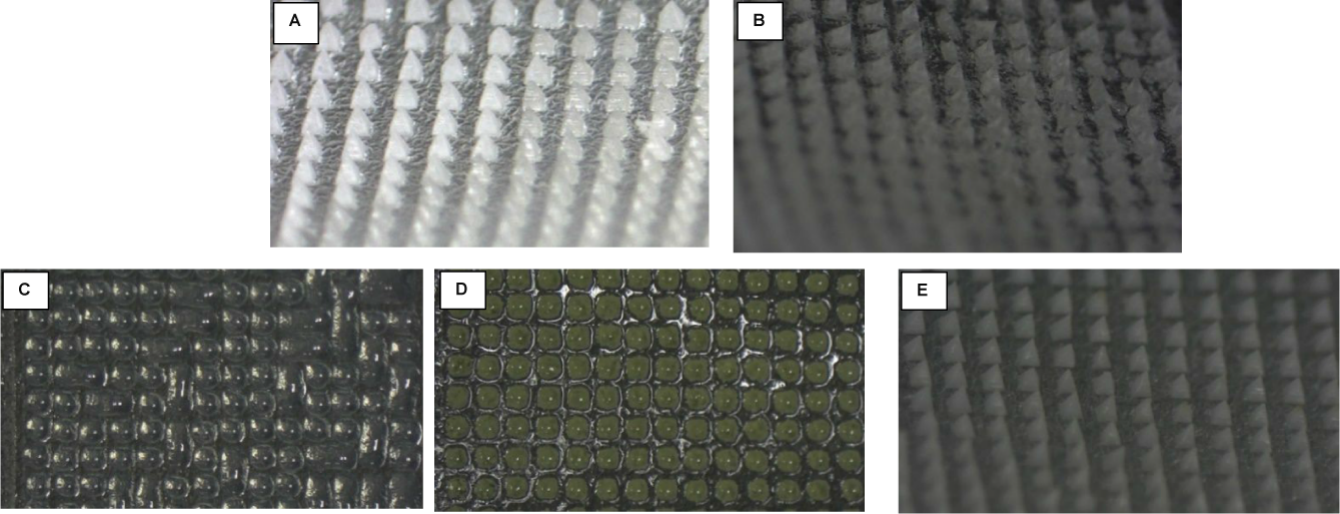
Shapes of the microneedles stored at 40 °C/75% RH on the day of instability visualized by an Olympus SZ61 microscope. (A) VDG array crystallized after 3 months; (B) BMD array crystallized after 1 month; (C) ZMT array melted after 1 day; (D) IMN array melted after 1 day; and (E) E2 array crystallized after 1 day.

**Figure 6:**
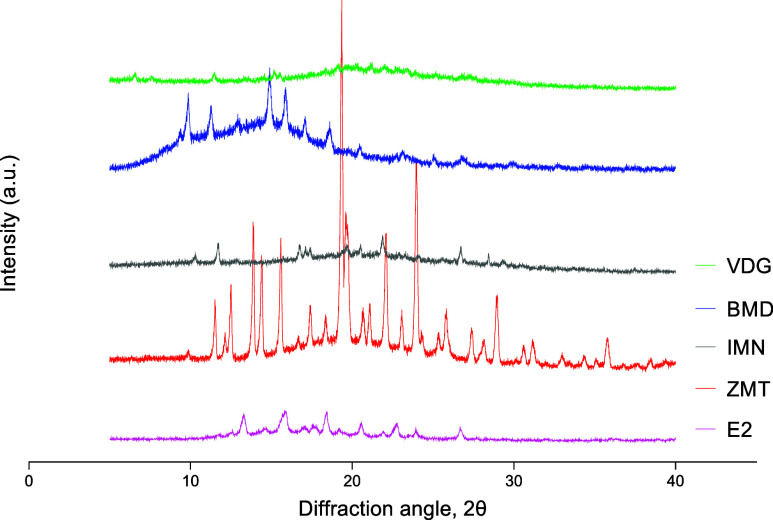
PXRD diffractograms of cooled-molten drug samples at the time point crystallization was first observed when stored at 40 °C/75% RH: VDG at month 3, BMD at month 1, and IMN, ZMT, and E2 on day 1 under 40 °C/75% RH.

At the 40 °C/75% RH storage condition, all drug microneedles and cooled-molten samples, except ITZ, exhibited instability. VDG microneedles crystallized into whitish needles by the 3-month time point, the BMD microneedles by one month, and the E2 microneedles after 24 h (Figure 5A,B,E). Meanwhile, IMN and ZMT microneedles melted on the first day of storage (Figure 5C,D). PXRD analysis confirmed that the corresponding drug samples exhibited crystallization at the same time points (Figure 6).

**Figure 7:**
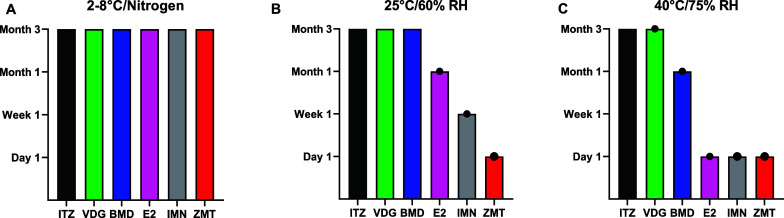
Amorphous stability of quench-cooled drug under storage conditions. (A) 2–8 °C with nitrogen; (B) 25 °C/60% RH; and (C) 40 °C/75% RH indicated by the first time point of crystallization or melting events were detected by DSC indicated by black circles.

The results clearly show that storage conditions significantly affect glass stability. At 2–8 °C with nitrogen, all samples retained their glassy state throughout the 3-month study, indicating optimal stability under these conditions. However, at 25 °C/60% RH, three drugs (E2, IMN, and ZMT) exhibited glass instability, with varying times to crystallization or melting. Increasing the storage temperature and humidity to 40 °C/75% RH resulted in instability for all drugs except ITZ, as summarized in Figure 7 and Table 1.

The experimentally determined glass stability of these drug substances was further investigated by comparing their physicochemical properties, specifically those associated with being good glass formers (Table 1). As all drug glasses were deemed to be stable when refrigerated under nitrogen, glass stability was ranked when stored under controlled humidity conditions. As a surrogate for molecular hydrogen bonding in this study, PSA was considered, which represents the combined surface area of oxygen, nitrogen, and hydrogen atoms bound to these electronegative atoms.^43^ With the highest *M*_w_ of 705.6 Da and PSA of 101 Å^2^, ITZ ranked as the most stable. For the six drugs investigated, the glass stability of the drug substances correlated with their *M*_w_ and PSA values for three of the drugs investigated. Following ITZ in stability was VDG, with an *M*_w_ of 421.3 Da and a PSA of 84.5 Å^2^, followed by BMD, which had an *M*_w_ of 504.6 Da and a PSA of 107 Å^2^. However, three drug substances, VDG, BMD, and E2, deviated from this pattern. Despite VDG and BMD both being categorized as class III GFA and BMD having a higher *M*_w_ and PSA compared to VDG, VDG exhibited slightly greater stability than BMD. Unexpectedly, E2, classified as a class II glass former, which possessed the lowest *M*_w_ and PSA (272.4 Da, 40.5 Å^2^), exhibited better glass stability than both class III ZMT and IMN.

It was assumed that water absorption by glassy drug microneedles increased molecular mobility, plasticizing the glassy state and inducing crystallization.^25^ To test this assumption, a second stability study was conducted in which ZMT and IMN microneedles were stored under a nitrogen atmosphere at 25 °C in sealed vials for 3 months, removing the effects of water vapor. ZMT and IMN microneedles were investigated as they exhibited the lowest difference between storage temperature (25 °C) and *T*_g_; ZMT (−24 °C) and IMN (−17 °C), and hence the plasticizing effect of humidity would have the greatest impact on glass stability.

**Figure 8:**
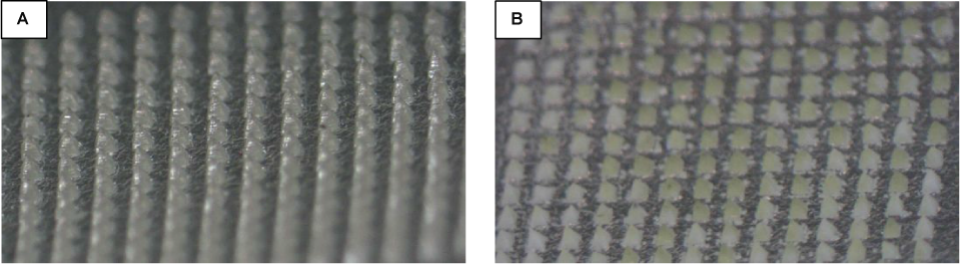
Shapes of microneedle patches stored at 25 °C/nitrogen visualized by an Olympus SZ61 microscope. (A) ZMT patch remained stable for 3 months. (B) IMN patch crystallized after a month and 3 weeks.

The results from this second stability study showed that IMN microneedles remained stable for 1 month and 3 weeks, after which crystalline whitish needles were observed (Figure 8B) compared to glass instability noted after 1 week at 25 °C/60% RH. In contrast, ZMT microneedles exhibited no signs of crystallization in the dry nitrogen environment at 25 °C throughout the entire 3-month study period (Figure 8A), demonstrating greater stability compared to their behavior at 25 °C/60% RH. These findings indicate that a dry nitrogen environment, eliminating the impact of humidity at 25 °C, can significantly enhance the stability of ZMT microneedles and somewhat increase the stability of IMN microneedles.

### Hygroscopicity of the Drug Substances

**Figure 9:**
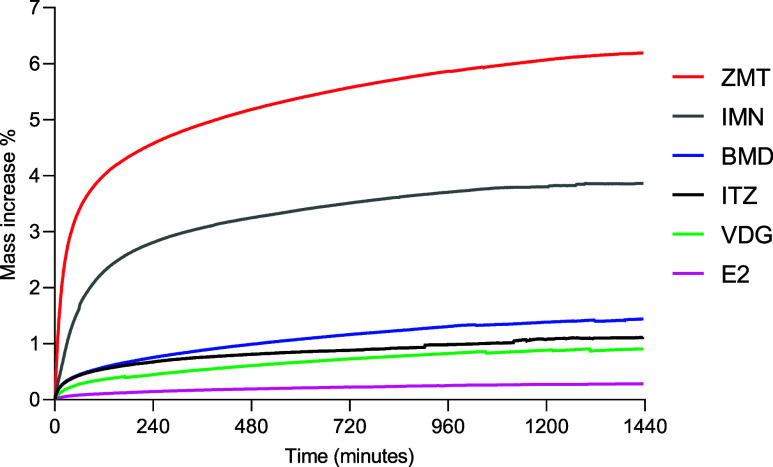
Increase in mass percentage moisture for each glassy drug form exposed to 80% RH at 25 °C for 24 h.

Glassy microneedle instability induced by water absorption plasticization observed for IMN and ZMT microneedles prompted investigation into the relative polarity of the glassy drug phases. As a measure of their polarity, their hygroscopicity was experimentally determined. Amorphous solids are more hygroscopic than their crystalline counterparts, meaning they can sorb moisture even at low humidity levels.^25^ The measured hygroscopicity of the drug glasses (Figure 9 and Table 1) can inform their relative glass stability under controlled humidity storage. The hygroscopicity of ITZ, VDG, and BMD was relatively low, with water uptake of 1.12%, 0.91%, and 1.44%, respectively. In contrast, ZMT, the least stable microneedle under humid conditions, exhibited the greatest hygroscopicity. ZMT fully melted/dissolved when stored at 40 °C/75% RH (Figure 5). IMN, the next least stable microneedle under humid conditions, exhibited the second highest hygroscopicity at 3.86%.

### Skin Penetration Assessment

**Figure 10:**
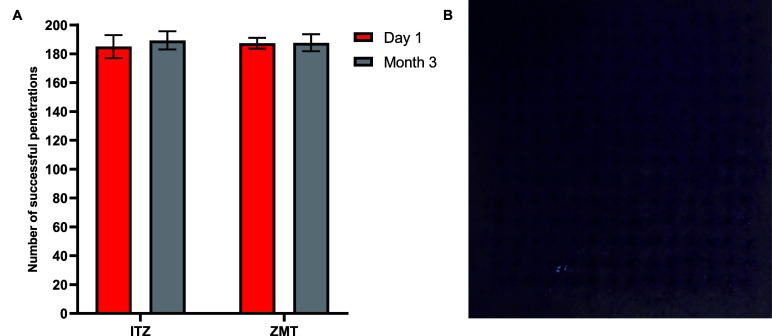
Ten skin penetration efficiency of ITZ and ZMT microneedle patches under different storage conditions. (A) Data represent the mean ± SD of the percentage of successful penetration for 14 × 14 microneedle patches, tested on the day of manufacturing and after 3 months of storage. ITZ was stored under 40 °C/75% RH, while ZMT was stored under refrigerated conditions under nitrogen. (B) Representative image of skin penetration showing stained perforations following ZMT microneedle application.

To confirm the skin penetration efficiency of glass microneedles stored under stabilizing conditions, ITZ and ZMT microneedles were evaluated before and after 3 months of storage. These microneedle arrays were selected as they represented the most and least stable glassy microneedles investigated (Table 1). The ITZ array was stored at 40 °C/75% RH, while the ZMT array was refrigerated and blanketed with nitrogen. Each microneedle array contained 196 (14 × 14) individual microneedle structures. Good skin penetration efficiency was determined post-fabrication and after 3 months of storage, with over 90% of the microneedle structures successfully penetrating the neonatal porcine skin model. No statistically significant differences were observed between the penetration efficiencies of ITZ and ZMT arrays or between arrays before and after storage (*p* > 0.05) (Figure 10). These results confirm that the microneedles retain their mechanical strength upon storage under conditions that maintain a glassy microneedle structure. These results align with previous findings for ITZ glassy microneedles fabricated as a 25 (5 × 5) microneedle array.^17^

## Discussion

In this study, we identified *T*_g_ and water absorption as the key properties that determine the glass stability of drug-only microneedles. Glass stability is critical for microneedle skin penetration. In agreement with previous studies,^19,24,42,44,45^ storing these glassy microneedles below their *T*_g_ was a key determinant of glass stability. At 2–8 °C, the *T*_g_ of all drugs investigated was ≥34 °C above the storage temperature, and all microneedles retained their glassy structure over the 3-month period investigated. This was further evidenced by the 3-month stability of ZMT microneedles stored at 25 °C (24 °C below ZMT *T*_g_) in a dry nitrogen environment. Microneedle glass stability when stored under dry conditions below their *T*_g_ values was in agreement with their GFA classification. However, it should be noted that due to the stability of all glasses investigated under this condition for the time period investigated, it was not possible to distinguish the relative stabilities of class II and class III compounds.

However, when glassy microneedles were stored under controlled humidity conditions (25 °C/60% RH and 40 °C/75% RH), glass stability was undermined for all drugs, except for ITZ. ITZ microneedles exhibited excellent glass stability at all storage conditions investigated. After 3 months of storage at 40 °C/75% RH, they retained their glassy appearance and skin penetration efficiency. Based on earlier studies demonstrating a relationship between glass stability and drug molecular weight,^20,45^ the stability of ITZ could be attributed to its high molecular weight relative to other drugs investigated. It has been suggested that the more complex structures of high molecular weight compounds render them less prone to forming an ordered crystalline state. However, it is important to consider how differences between the ITZ glass structure and the other drug glasses affect ITZ microneedle stability on storage. The ITZ glass formed upon cooling of its melt contains liquid crystal phases, as evidenced by thermotropic mesophase transitions (Figure 1) and ITZ microneedle whitish appearance (Figure 2).^40,41^ Its liquid crystal glass structure would contribute to the superior ITZ microneedle stability when stored under controlled humidity. A study by Mugheirbi et al. demonstrated how the crystallization of glassy ITZ was inhibited when stored under humidity at 50 °C (below its *T*_g_) due to the formation of more ordered liquid crystal phases via ITZ complexation with water.^46^ This interaction between ITZ glass and absorbed water deviates from the more commonly reported plasticizing effect of absorbed water in glassy materials.^25^ It is important to note that water absorption by drug glass phases does not always result in plasticization and that plasticization is dependent on the intermolecular interactions between the specific glass and water molecules. For example, the glass form of prilocaine exhibited antiplasticization and a reduction in molecular mobility in the presence of absorbed water, which was attributed to the formation of strongly hydrogen-bonded prilocaine–water dimers or complexes.^47^

For GFA class III molecules, VG and BMD, glassy microneedles were stable at 25 °C/60% RH for 3 months but recrystallized when stored at 40 °C/75% RH at 3 months and 1 month, respectively. These results illustrate opposing influences on molecular mobility, a key determinant of glass stability. Molecular mobility is reduced due to storage at temperatures below the *T*_g_, while molecular mobility is increased due to water absorption inducing glass plasticization.^25^ At 25 °C, the difference in temperature between storage and *T*_g_ (dry), VG (−59 °C) and BMD (−71 °C), is sufficiently large that a reduction in Tg due to the plasticizing affects moisture absorption at 60% RH does not undermine glass stability. However, at 40 °C/75% RH, the margin between storage and *T*_g_, VG (−44 °C) and BMD (−51 °C), was not sufficient to offset the plasticizing affects moisture absorption at 75% RH. A probable cause of glass instability was the reduction of *T*_g_ due to plasticization in the region of, or below, the storage temperature facilitating crystallization.

Microneedle instability was greatest for IMN and ZMT microneedles under controlled humidity. These IMN and ZMT amorphous forms exhibited the greatest hygroscopicity and lowest *T*_g_ values, IMN (42 °C) and ZMT (49 °C), of the drug investigated. The instability of these glasses is attributed to the plasticization effect of water. The stability of these glassy microneedles was greatly increased at 25 °C when environmental humidity was eliminated. ZMT microneedles retained their glassy structure after 3 months of storage in a dry nitrogen environment at 25 °C. The instability of IMN microneedles stored below IMN *T*_g_ agrees with previous reports of IMN crystallization at temperatures below *T*_g_ in the presence and absence of humidity.^48,49^

E2 was the only GFA class II compound investigated. E2 microneedles crystallized under both 25 °C/60% RH and 40 °C/75% RH at 1-month and 1-day time points, respectively. In comparison to VG, a GFA class III drug with a similar *T*_g_ (Table 1), E2 was less stable in controlled humidity environments (Table 1). In this respect, the GFA class appeared to inform their relative stabilities under storage, with class III molecules being more stable than class II. However, the relationship does not extend to all class III molecules investigated. E2 microneedles displayed greater stability at 25 °C/60% RH than ZMT and IMN microneedles. E2’s greater stability can be related to its lower hygroscopicity and higher *T*_g_ relative to ZMT and IMN.

Based on this experimental study with a limited set of six molecules, it was challenging to identify how molecular properties relate to glass stability across the storage conditions investigated. GFA class was a good predictor of glass stability below *T*_g_ when stored under “dry” conditions. While molecular descriptors can predict GFA,^21,45,50^ crystallization tendency requires experimental determination. Currently, long-term predictions of crystallization during isothermal studies below *T*_g_ are challenging even with experimentally acquired data.^50^ Employing a larger data set of 130 molecules, a computational model to predict crystallization tendency based on calculated molecular descriptors alone was developed.^51^ The measure of crystallization tendency used to develop the model was the experimentally determined GFA class.^20^ The model developed distinguished a combined set of GFA class II and III molecules from class I molecules. However, discriminating class II molecules from class III molecules was challenging. As previously reported^25,51^ and observed from the results of this study, glass stability is dependent on external factors including storage temperature and glass interaction with moisture vapor. Therefore, inclusion of these factors and their interaction with intrinsic molecular descriptors require consideration in the development of models to predict glass crystallization tendency.

Study findings provide insights into how drug-only glassy microneedles can be stabilized during storage by elucidating key interactions between glassy microneedle properties and storage conditions. These insights are critical for optimizing packaging and storage conditions that maintain the microneedle mechanical properties and skin penetration efficacy throughout shelf life. Results show that by stabilizing the glassy microneedle structure via the selection of suitable storage conditions, microneedle skin penetration efficiency can be maintained (Figure 10). While this research was focused on glassy microneedle formulations, it also has relevance for glassy drug structures incorporated in oral dosage forms, coatings on medical devices, and dry powder inhaled drug formats.

## Conclusion

In this study, we identified *T*_g_ and water absorption as the key properties that determine the glass stability of drug-only microneedles. The study findings highlight that GFA classification can predict microneedle glass stability when stored under dry conditions below *T*_g_. However, this relationship is undermined when glass microneedles are exposed to humidity during storage. Under humid conditions, microneedle stability was influenced not only by glass hygroscopicity but also by the plasticizing effect of the absorbed water. The storage stability of ZMT microneedles at 25 °C was increased from 1 day under 60% RH to 3 months under nitrogen. In contrast, ITZ microneedles maintained their glass structure when stored at 40 °C/75% RH. The superior stability of ITZ microneedles when exposed to humidity was related to their liquid crystalline glass structure. The study findings also demonstrate how glassy microneedle skin penetration efficiency is maintained during storage by selecting storage conditions that stabilize their glassy structure.

## Supporting Information Available

DSC and PXRD results for cooled-molten samples of itraconazole, vismodegib, betamethasone dipropionate, indomethacin, zolmitriptan, and estradiol after 3 months storage at 2–8 °C/nitrogen (PDF)
