# Supplementary material for: Glassy Drug Microneedle Array Design: Drug Glass-Forming Ability and Stability
Source: Mol Pharm. 2025 Feb 17;22(3):1373–83. doi: 10.1021/acs.molpharmaceut.4c01067 (PMC11881139; doi:10.1021/acs.molpharmaceut.4c01067)
Supplement: Supplementary file 1 — mp4c01067_si_001.pdf [file mp4c01067_si_001.pdf]

Supporting Information: Glassy drug microneedle array design: Drug glass forming ability and stability

Mohamed Elkhashab[1], Ziad Sartawi[2], Waleed Faisal[2], Abina Crean[1]*

[1] SSPC, the Research Ireland Centre for Pharmaceutical, School of Pharmacy, University College Cork, Cork, T12 K8AF, Ireland

[2] ArrayPatch Ltd, Euro Business Park, Little Island, Cork, T45 FX94, Ireland

[*] Email: a.crean@ucc.ie

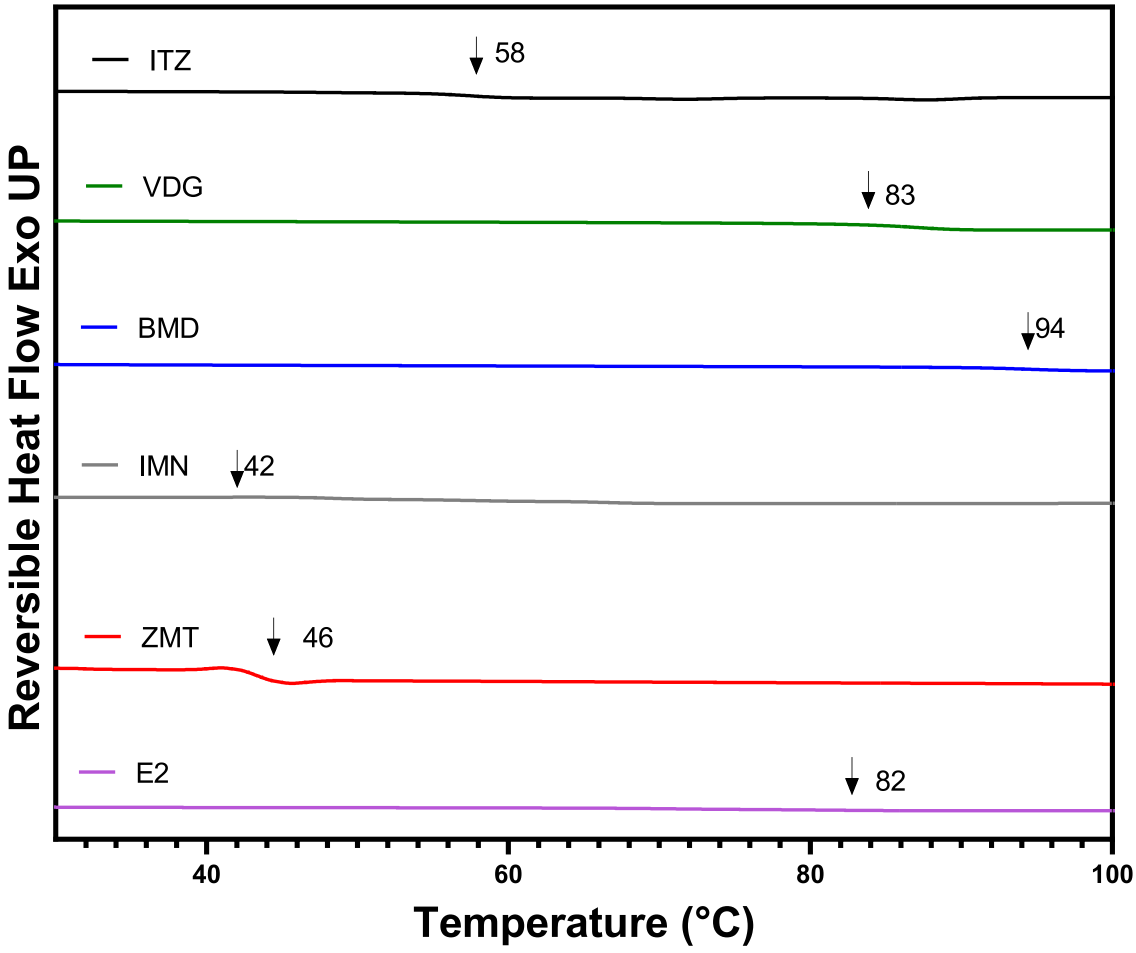

Figure S1: DSC thermogram of the cooled-molten samples of itraconazole (ITZ), vismodegib (VDG), betamethasone dipropionate (BMD), indomethacin (IMN), zolmitriptan (ZMT) and estradiol (E2) after 3 months storage at 2-8°C/nitrogen with glass transition temperatures indicated

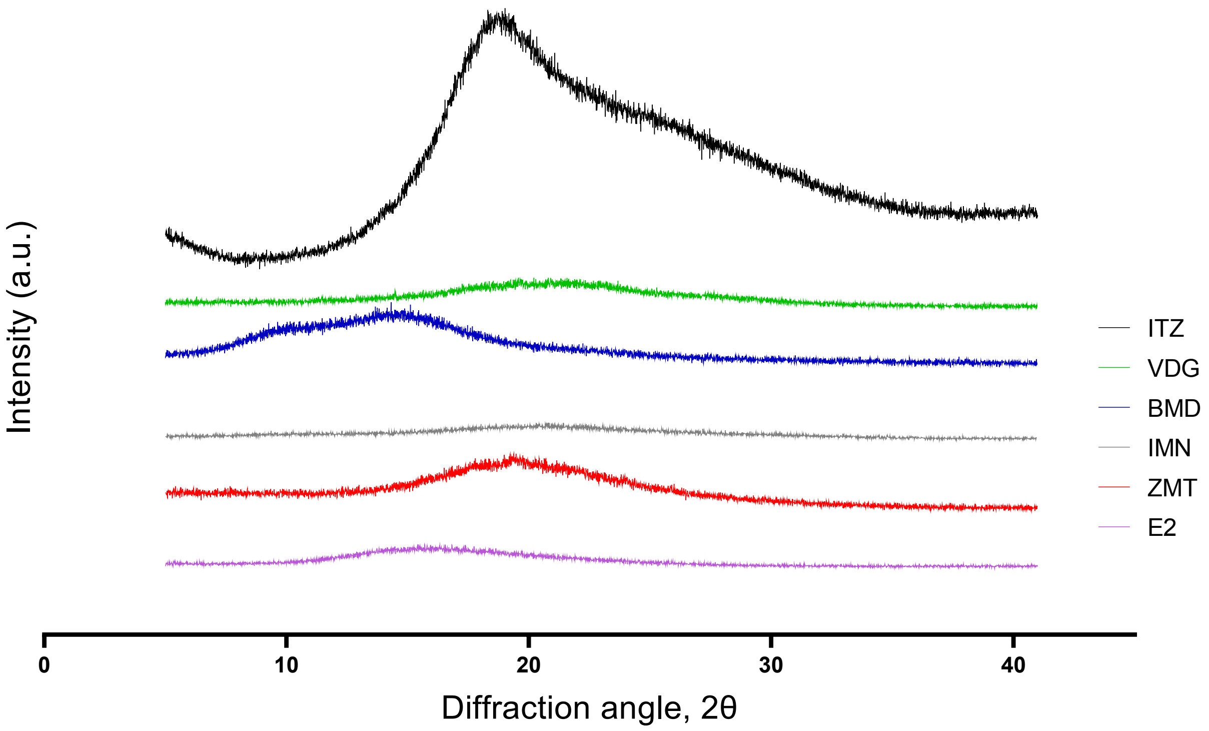

Figure S2: PXRD diffractogram of the cooled-molten samples of itraconazole (ITZ), vismodegib (VDG), betamethasone dipropionate (BMD), indomethacin (IMN), zolmitriptan (ZMT) and estradiol (E2) after 3 months storage at 2-8°C/nitrogen.
